# Does Exercise Improve Glycaemic Control in Type 1 Diabetes? A Systematic Review and Meta-Analysis

**DOI:** 10.1371/journal.pone.0058861

**Published:** 2013-03-15

**Authors:** Amy Kennedy, Krishnarajah Nirantharakumar, Myriam Chimen, Terence T. Pang, Karla Hemming, Rob C. Andrews, Parth Narendran

**Affiliations:** 1 School of Clinical & Experimental Medicine, University of Birmingham, Birmingham, United Kingdom; 2 Department of Diabetes, University Hospitals Birmingham NHS Foundation Trust, Birmingham, United Kingdom; 3 Public Health, Epidemiology & Biostatistics, University of Birmingham, Birmingham, United Kingdom; 4 Department of Diabetes & Endocrinology, Dudley Group of Hospitals NHS Foundation Trust, Dudley, United Kingdom; 5 School of Clinical Science, University of Bristol, Bristol, United Kingdom; 6 Department of Diabetes & Endocrinology, Taunton & Somerset NHS Foundation Trust, Taunton, United Kingdom; Children’s Hospital Boston, United States of America

## Abstract

**Objective:**

Whilst regular exercise is advocated for people with type 1 diabetes, the benefits of this therapy are poorly delineated. Our objective was to review the evidence for a glycaemic benefit of exercise in type 1 diabetes.

**Research Design and Methods:**

Electronic database searches were carried out in MEDLINE, Embase, Cochrane’s Controlled Trials Register and SPORTDiscus. In addition, we searched for as yet unpublished but completed trials. Glycaemic benefit was defined as an improvement in glycosylated haemoglobin (HbA1c). Both randomised and non-randomised controlled trials were included.

**Results:**

Thirteen studies were identified in the systematic review. Meta-analysis of twelve of these (including 452 patients) demonstrated an HbA1c reduction but this was not statistically significant (standardised mean difference (SMD) −0.25; 95% CI, −0.59 to 0.09).

**Conclusions:**

This meta-analysis does not reveal evidence for a glycaemic benefit of exercise as measured by HbA1c. Reasons for this finding could include increased calorie intake, insulin dose reductions around the time of exercise or lack of power. We also suggest that HbA1c may not be a sensitive indicator of glycaemic control, and that improvement in glycaemic variability may not be reflected in this measure. Exercise does however have other proven benefits in type 1 diabetes, and remains an important part of its management.

## Introduction

The current UK guidelines for exercise and physical activity are that all individuals undertake at least 150 minutes of moderate-intensity, or 75 minutes of high-intensity, aerobic physical activity per week. [1] The American Diabetes Association (ADA) has similar recommendations for all people with diabetes. [2] The evidence base for these recommendations for patients with diabetes is drawn largely from studies of the general population and those with type 2 diabetes. [3] Studies on the benefits of exercise in type 1 diabetes are less robust.

The currently recognised health benefits of exercise in type 1 diabetes have been reviewed previously. [3] To date, there is evidence that exercise improves physical fitness, insulin resistance, lipids and macrovascular risk in people with type 1 diabetes. [3] However, the results of studies on exercise and glycaemic control are conflicting. It is important to clarify this issue because glycaemic control is considered by both patients and health care professionals to be the mainstay of type 1 diabetes management. Furthermore, and in contrast to studies in type 1 diabetes, a variety of different forms (aerobic/resistance) of exercise have a demonstrable benefit on glycaemic control in type 2 diabetes [4], [5].

Here, we perform a systematic review and meta-analysis of randomised controlled and non-randomised parallel group trials of exercise training and glycaemic benefit in type 1 diabetes. We use glycosylated haemoglobin (HbA1c) as the measure of glycaemic control.

## Methods

### Data Sources and Searches

The electronic databases searched were MEDLINE (Ovid), Embase (Ovid), Cochrane library (Wiley) and SPORTDiscus (*EBSCO*) up until August 2011. An updated search of Medline was performed up until May 2012. A variety of search terms were used for individuals with type 1 diabetes (population) and physical activity (intervention). We did not restrict our search to the outcome or study design to maximise the sensitivity of our search. Nor did we place any date or language restrictions. Our secondary search strategy included scanning bibliographies of the retrieved articles and searching for unpublished studies using key trial registries (clinicaltrials.gov, Current Controlled Trials Registry, WHO hosted International Clinical Trials Registry Platform - ICTRP). The primary search strategy is outlined in the Appendix S1. The systematic review and meta-analysis is reported according to the Preferred Reporting Items for Systematic Reviews and Meta-Analyses (PRISMA) guidelines [6].

### Study Selection

Inclusion criteria were defined based on population, intervention, comparator, outcome and study design. Population were adults and children with type 1 diabetes. Where the studies reported data for both type 1 and 2 diabetes, authors were contacted to obtain data for type 1 diabetes alone. Interventions that aimed to increase the exercise through either supervised or unsupervised training were included. We did not exclude studies based on the intensity or type of exercise. Only trials that involved a non-intervention group of participants with type 1 diabetes were included. Studies where any other intervention was given to the participants (e.g. dietary intervention) were only included if they were given to both intervention and control arms. We included both randomised and non randomised parallel controlled trials.

The primary outcome measure was change in HbA1c, with HbA1c data extracted before and after the intervention. In those studies where complete HbA1c data was not reported, we contacted the authors for clarification. Our secondary outcome measure was adverse events (hypoglycaemia).

### Data Extraction and Study Quality

Initial selection was based on title and abstract. The original article was obtained where it was unclear whether the study met our inclusion criteria. Two reviewers (AK and MC) selected the studies independently. Where there was disagreement between the two reviewers, resolution was through a meeting with PN and KN. Reasons for exclusion were documented and are available from the authors on request. Where necessary, foreign language papers were translated.

A data extraction and quality assessment form was developed based on the template suggested by Centre for Review and Dissemination (CRD) for systematic reviews in their guidelines [7] Data extraction was performed by AK and checked by KN for accuracy and for any missing information. Extracted data included details of the study population, intervention characteristics, outcome measures, and areas of potential bias. Biases evaluated in randomised controlled trials (RCT) included adequate sequence generation, satisfactory allocation concealment, follow-up and exclusion biases, blinding of outcome assessors and intention to treat analysis. In non RCTs we evaluated any baseline differences in participants, biases in allocation, follow-up and exclusion and if analytical method was specified in the study protocol.

### Data Synthesis and Analysis

Each of the trials measured the outcome (HbA1c) both before and after the intervention period, and for both control and intervention arms. Trials did not report paired difference summary statistics and so we could not obtain paired difference measures. Rather, it was necessary to extract information on mean, standard deviations, and number of participants both before and after the intervention period and for both intervention and control arms for each trial. For trials which reported the standard error of the mean (SEM), we converted this to a standard deviation (SD) by multiplying by the square root of the number of participants in that arm. Any studies that only reported median values without any measure of variation (such as inter-quartile range) could not be included in the analysis.

For any trials which were multiple intervention arm trials we extracted and summarised the trial information for each arm. We then combined the intervention arms into a single combined arm by estimating the weighted mean values (both before and after) and estimating the weighted SD using the usual pooled standard SD [9]. A similar approach was taken where there were two control arms.

From the estimated mean and SD values for HbA1c both before and after the intervention period, we then estimated the mean difference (after-before) and SD in both the control and intervention arms. It was necessary to estimate the SD for these change effects as no estimates of correlation (or SDs of changes) were reported in the original trial reports. We used a conservative estimate for the correlation (0.5) as advocated [8].

All trials reported on the outcome HbA1c. However, because HbA1c-DCCT standardisation only occurred in 1997, we summarised treatment effects using the standardised mean difference (SMD) of Hedges adjusted g [9].

Clinical, methodological and statistical heterogeneity were explored. Clinical heterogeneity was explored by considering the clinical diversity of the study participants in terms of their age and study duration. Methodological heterogeneity was explored by exploring variations in randomised and non randomised studies. Statistical heterogeneity was tested using Cochran’s Q, a statistic based on the chi-square test and quantified by the I-square test. Results were then pooled across studies using a random effects meta-analysis (since the degree of heterogeneity was too large to warrant a fixed effects analysis) using the generic inverse variance method. We pooled over all studies within the subgroups of child and adult studies. We further investigated the effect of age and duration of intervention by including the pooled (over control and intervention arms) weighted mean age and duration as covariates in a meta-regression model. Residual levels of heterogeneity were investigated by comparing I-squared values before and after adjustment for covariates (age and duration).

Risk of small study bias (or publication bias) was explored using the contour enhanced funnel plot, stratifying by age of study population (adult or child) and was further evaluated by the Egger test.

## Results

**Figure 1:**
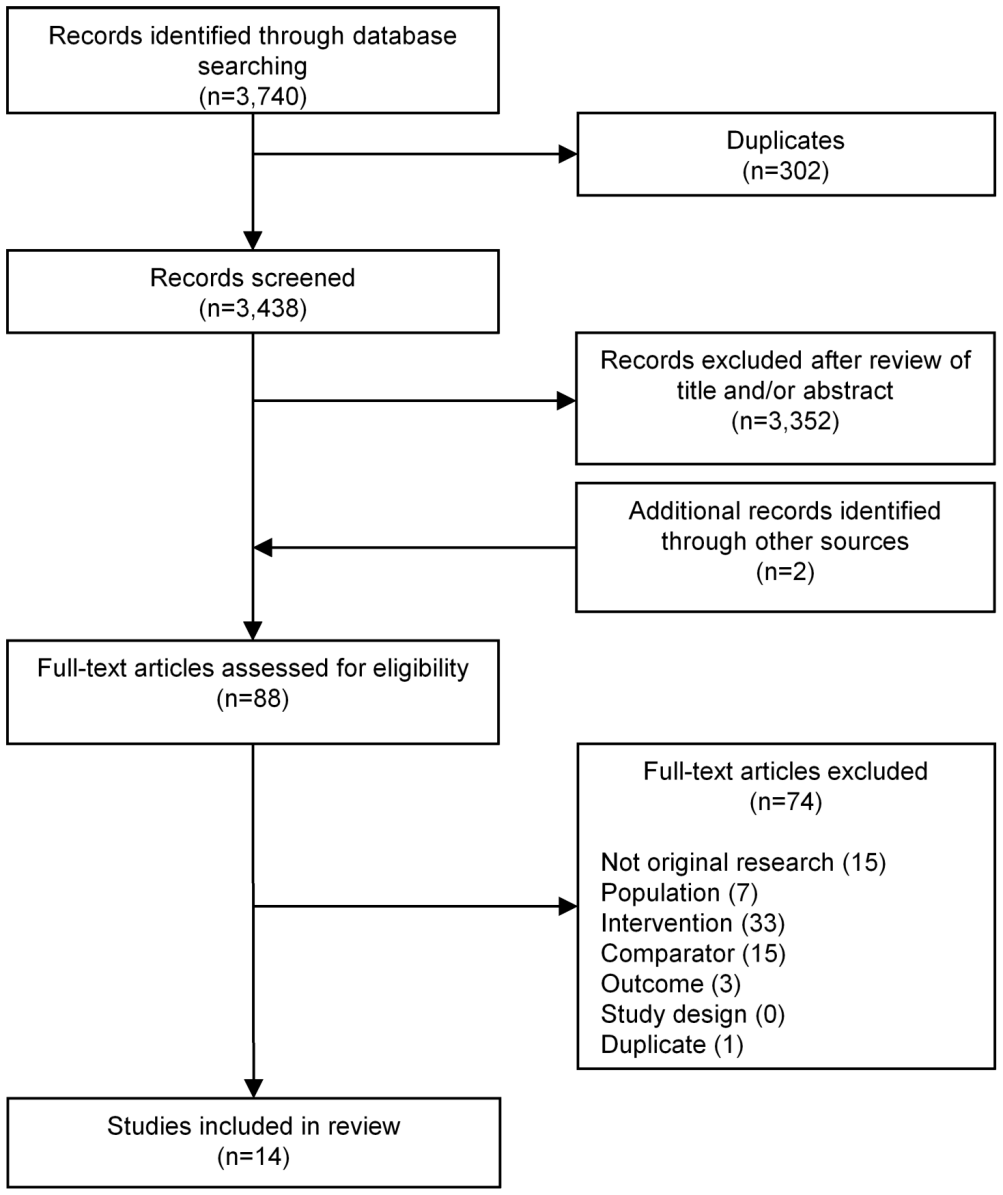
Flow diagram of articles identified.

The database searches identified a total of 3,740 potentially relevant papers (figure 1). One further paper was identified through follow-up of a conference abstract (identified in the original searches). After removing duplicates and screening by considering titles and abstracts, 94 full-text articles were assessed. Of these, 14 studies met the pre-determined inclusion criteria. One study [10] did not report separate results for subjects with type 1 diabetes and these were not available after contacting the authors. Hence, thirteen studies were considered suitable for inclusion in the systematic review. The trials included both adults and children (Table 1), with nine of the 13 trials recruiting children and young adults (under 18 years of age). Many studies involved only aerobic exercise (9/13), three studies involved both aerobic and resistance activities, and one did not specify the exercise type.

**Table 1:** Study Characteristics and Results.

Source	RCT Y/N	Intervention	Weekly duration, min	Program duration	Adherence to Exercise training, %	Age, Mean, yrs		Number of participants analysed	HbA1c (mean,% unless stated otherwise)			
									Baseline		At end of training period	
						Control group	Training group		Control group	Training group	Control group	Training group
**Children and adolescents**												
Dahl-Jorgensen et al, 1980 [24]	N	Supervised exercise	120	5 m	not stated	11		22	13.4	15.1	12.9	13.8
Campaigne et al, 1984 [16]	Y	Supervised high-intensity aerobic exercise	90	12 w	>75%	8.5	9.0	19	13.9	12.5	13.3	11.3
Landt et al, 1985 [14]	Y	Supervised high-intensity exercise	135	12 w	not stated	15.9	16.1	15	12	12	12	12
Huttunen et al, 1989 [29]	Y	Supervised (high intensity) aerobic exercise	60	13 w	88% (11.5/13)	11.9		32	9.4	9.8	9.7	10.5
Heyman et al, 2007 [30]	Y	Supervised high intensity aerobic and resistance exercises c	120+60^a^	6 m	62–100%^a^	16.3	15.9	16	8.5	7.3	8.2	7.1
Salem et al, 2010 [15] **(**less frequent intervention)	Y^b^	Supervised (high intensity)aerobic and resistance exercises once/week	30	6 m	100%^b^	15	14.7	123^c^	8.3	8.9	8.9	7.8
Salem et al, 2010 [15](more frequent intervention)	Y^b^	Supervised (high intensity) aerobic and resistance exercises 3x/week	90	6 m	100%^b^	15	14.5	121^c^	8.3	8.9	8.9	8.1
Aouadi et al, 2011 [22] (lower duration intervention)	N	Supervised aerobic exercise for 2 hours/week	120	6 m	not stated	12.9	12.2	22^c^	9.6	8.8	9.8	8.2
Aouadi et al, 2011 [22](higher duration intervention)	N	Supervised aerobic exercise for 4 hours/week	240	6 m	not stated	12.9	13.5	22^c^	9.6	8.2	9.8	6.8
Wong et al, 2011[31]	N	Unsupervised aerobic exercise	approx 90	12 w	>67%	12.98	11.62	28	8.40^d^	8.06	8.41^d^	7.80
**Adults**												
Yki-Jarvinen et al, 1984 [11]	N	Supervised (high intensity) aerobic exercise	240	6 w	not stated	24	26	13	8.4	8.6	9.2	8.9
Wallberg-Henriksson et al, 1986 [12]	Y^e^	Supervised aerobic exercise. High intensity in final 2/12	140	5 m	74%(5.2/7)	35	36	13	10.6	10.4	10.4	10.5
Laaksonen et al, 2000 [20]	Y	Supervised aerobic exercise	120+	12–16 w	Not stated.	29.5	20	42	8.3	8.2	8.5	8.0
Fuchsjager-Mayrl et al, 2002 [23]	N	Supervised aerobic exercise	150+^f^	4 m	81%	33	42	23	7.4	7.3	7.4	7.5
**Excluded**												
De et al, 2011 Do we D’Hooge 2011 [13]	Y	Supervised moderate –intensity aerobic and resistance exercises	140	20 w	63% (24/38)	13.2	14.1	16	8.8^g^	7.9^g^	8.6^g^	7.8^g^

a Participants performed one 2 hour supervised exercise session and an additional one 1 hour unsupervised session per week. Attendance for the unsupervised session was 52–89%.

b Although reported as a RCT, groups were randomly assigned by a diabetologist to either exercise programme. It is not clear if the controls were selected randomly or those who didn’t attend the exercise session were designated as controls.

c Control participants are the same for both interventions. These participants therefore appear twice in this table, explaining a total of 452 participants across all studies in this table.

d Weighted mean (for two control groups).

e Randomisation occurred before consent was sought.

f Participants could undertake an additional unsupervised exercise session at home.

g Median.

Of the thirteen included studies, eight were randomised controlled trials. Randomisation procedures were not reported in any of these studies. In one study the reported randomisation method (alternate subjects assigned to each group) resulted in it being classified as non-randomised parallel group trial for the purposes of this review. [11] In one study [12], the randomisation process was carried out prior to consent being sought. Allocation concealment and blinding were only reported in one study. [13] Losses to follow-up and exclusions were documented in all studies. Of the eight RCTs included in this study, five were analysed according to the intention to treat principle. Four out of the five non-randomised parallel group trials used a protocol based analysis. Adherence to the training programme was not reported in five studies. Where it was reported, adherence ranged between 62 and 100%. ‘Quality’ measures of the included studies are listed in Table 2.

**Table 2:** Quality Assessment.

Source	RCTY/N	Is there a descriptionof the sequence generation for randomisation?	Is the allocation concealment described?	Were the outcome assessors blinded?	Was the analysis done on an ‘intention-to-treat’ basis (or protocol-based for Non RCT Study)	Controls	Dropouts, n	Exclusions, n
Dahl-Jorgensen et al, 1980[24]	N	N/A	N/A	N	N	Similar for age and disease duration	0	0
Campaigne et al, 1984[16]	Y	N	N	N	Y	Similar for age, weight, height, disease duration, insulin dose and HbA1c	0	0
Landt et al, 1985 [14]	Y	N	N	N	N	Groups similar for age, weight, disease duration and insulin dose	0	1
Huttunen et al, 1989 [29]	Y	N	N	N	N	Age- and sex-matched pairs, similar baseline fitness, BG and HbA1c	2	0
Heyman et al, 2007 [30]	Y	N	N	N	Y	Groups similar for age, weight, BMI,disease duration, baseline physical activity and insulin dose	0	0
Salem et al, 2010[15]	Y^b^	N	N	N	N	Similar for HbA1c, age, hypos, BMI, waist, insulin doses. Higher LDL C, TG, chol and duration of DM in 3x/week group	46	0
Aouadi et al, 2011 [22]	N	N/A	N/A	N	N	Similar for age, BMI and diabetesduration.	0	0
Wong et al, 2011 [31]	N	N/A	N/A	N	N	Patients not given exercise video/andnot given extra calls to encourageadherence	4	9
Yki-Jarvinen et al, 1984 [11]	N	N/A	N/A	N	N	Sedentary patients on CSII controls	0	0
Wallberg-Henriksson et al, 1986 [12]	Y^c^	N	N	N	N	Similar for age, BMI, duration ofdiabetes, previous activity levels. Lower insulin dose in intervention group	6	2
Laaksonen et al, 2000 [20]	Y	N	N	N	Y	Similar for age, fitness, insulin dose,HbA1c, lipids	14	0
Fuchsjager-Mayrl et al, 2002 [23]	N	N/A	N/A	N	N	Controls exercised more than onceweekly at baseline (Interventiongroup were sedentary)	3	0
D’Hooge et al, 2011 [13]	Y	N	Y	Y	Y	2 CSII in intervention group, none in control, unchanged over study period. Similar for insulin dose, age, disease duration, BMI, waist circ	0	0

b Although reported as a RCT, groups were randomly assigned by a diabetologist to either exercise programme. It is not clear if the controls were selected randomly or those who didn’t attend the exercise session were designated as controls.

c randomisation occurred before consent was sought.

### Exercise and HbA1c

The study by D’Hooge et al [13] could not be included in the meta-analysis because median HbA1c without interquartile range was reported. This study reported a 0.1% fall in median HbA1c in the intervention group and a 0.2% fall in control group with exercise.

**Figure 2:**
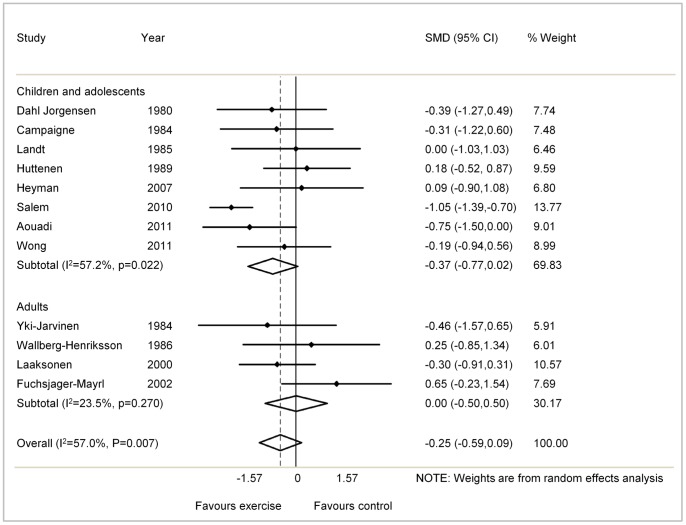
Results of studies of physical activity and glycaemic control (HbA1c) in type 1 diabetes patients according to age groups of participants.

The remaining 12 studies (452 patients) were included in the meta-analysis. The overall, pooled data showed a non-significant SMD reduction in HbA1c of −0.25% with exercise training (95% CI, −0.59 to 0.09; p = 0.144; I-squared = 57.0%, Figure 2). A greater SMD reduction in HbA1c was seen in the eight studies of children and young adults, but here also this was not significant (SMD, −0.37; 95% CI, −0.77 to 0.02; p = 0.066; I-squared = 57.2%). No effect was seen in adult studies, of which there were four, (SMD, 0; 95% CI, −0.5 to 0.5; p = 0.998; I-squared = 23.5%).

In sensitivity analysis, there was no association between study design and HbA1c reduction: SMD for randomised studies was −0.24 (95% CI −0.71 to 0.23) and for non-randomised studies −0.25 (95% CI −0.59 to 0.09). The degree of heterogeneity observed in the randomised studies was I-squared = 66.4% whereas this was lower in the non-randomised studies (I-squared = 33.4%).

**Figure 3:**
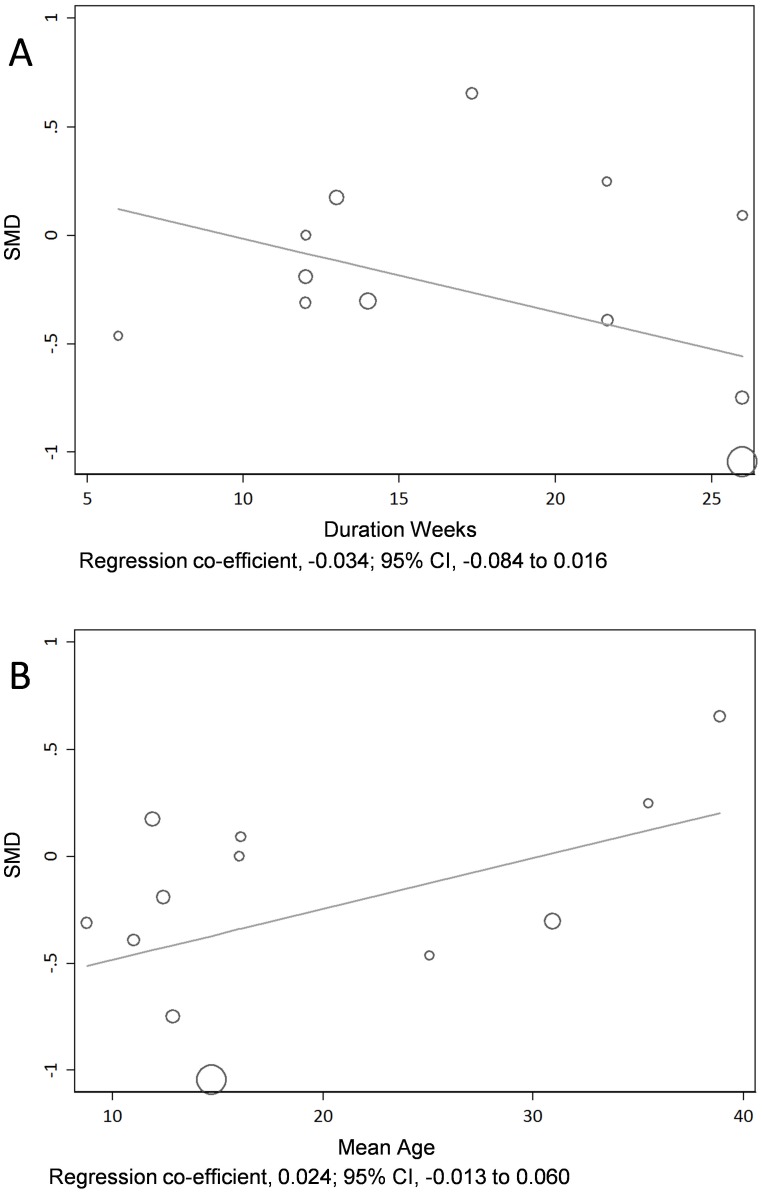
Metaregression by duration of study (A) and age of participants (B).

Meta regression of HbA1c against length of study duration suggested a non-significant association between HbA1c reduction and longer intervention (regression co-efficient, −0.034; 95% CI, −0.084 to 0.016; p = 0.163, figure 3A). Similarly, there was a non-significant association of HbA1c and increasing age (regression co-efficient, 0.024; 95% CI, −0.013 to 0.060; p = 0.179, figure 3B). The I-squared value before adjustment for co-variates was 57%, and reduced to 50.4% after adjustment for age; and to 34.2% after adjustment for duration.

The funnel plot (figure S1) suggests evidence of small study or publication bias with under representation of studies reporting negative effects (Eggers test p value = 0.003).

### Exercise and Adverse Events

Hypoglycaemia frequency was mentioned in only five studies. [11], [13]–[16] One study [14] reported a similar frequency of hypoglycaemia in both the intervention and control group. Campaigne et al [16] reported only one episode of hypoglycaemia, which occurred during a training session. Salem et al [15] found no difference in hypoglycaemia rates between the groups exercising once or three times per week, but did not comment on the hypoglycaemia frequency in the control group. Conversely, Yki-Jarvinen et al [11] found that hypoglycaemia rates were increased in the first two weeks of the training program but diminished thereafter, and D’Hooge et al [13] reported frequent hypoglycaemia episodes both during and after exercise in the intervention group.

## Discussion

This meta-analysis does not reveal a glycaemic benefit of exercise in people with type 1 diabetes. However, sub-analyses suggests that exercise may confer a glycaemic benefit in the young, and when undertaken for longer periods. Whilst adverse event reporting was poor, these studies demonstrate that exercise can be undertaken by people with type 1 diabetes without significant hypoglycaemia. This is important because qualitative studies have previously reported hypoglycaemia to be a barrier to exercise in type 1 diabetes [17], [18].

We used predetermined inclusion criteria designed to identify as many relevant studies as possible, and conducted this analysis in accordance with PRISMA guidelines. [6] However, this study is limited by the availability and quality of the obtained data. We were unable to obtain the necessary data from all identified trials, either because the data was not presented in a way that enabled extraction for analysis or because we were unable to contact the authors of the study. There is evidence from our analysis that studies showing a negative effect of exercise on glycaemia may not have been published (or identified during our search of unpublished trials), and so that the effect size shown in this meta-analysis is possibly overestimated.

There was considerable variation in the intervention (form and intensity of exercise, duration of intervention), which may have contributed to the heterogeneity of study results. However, ADA guidance on exercise for people with type 1 diabetes is limited only to duration and intensity [2], therefore, inclusion and comparison of studies with different exercise interventions is, in our opinion valid.

There are a number of potential explanations for the findings of this meta-analysis. Firstly, the programmes of exercise may not have been of sufficient duration. This is supported by our sub-analysis which shows a trend for HbA1c reduction with longer duration of intervention. Based on pooled data from these studies, and assuming that the rate of glycaemic benefit persists in a linear fashion, we estimate that studies of greater than 25 weeks duration would be needed to obtain an HbA1c reduction in the region of 0.5%. This has implications for the design of future trials. Secondly, as has been demonstrated in some studies of type 2 diabetes, [19] the intensity of the exercise program may be important. Inadequate reporting of exercise intensity in the current type 1 diabetes studies makes this difficult to analyse. Increased calorie intake, either as a source of fuel to manage hypoglycaemia or as a reward, is another possible reason why our analysis failed to detect a glycaemic benefit of exercise. The interventions in the current studies were associated with additional carbohydrate intake, and this is in line with ADA guidance as a means of avoiding hypoglycaemia. However diet in general was poorly recorded in these studies. Laaksonen et al [20] did attempt to record dietary intake. In those participants where this was achieved, dietary intake appeared similar between training and control groups. There is therefore a need for studies in which dietary intake is controlled for, or calorie intake clearly recorded. There have been studies of combined diet and exercise interventions [21] which were excluded from our analysis as the dietary advice was given to the training arm alone. This study did show an HbA1c reduction with combined dietary and physical activity (8.9±2.6 to 8.6±2.1% vs 8.7±2.0 to 8.8±2.3% in the control arm), although it did not reach significance. Insulin dose adjustment is another approach to avoiding hypoglycaemia around exercise, and a reduction in insulin dosage may account for the absence of a reduction in HbA1c. Of the 11 studies that reported insulin dosage pre- and post-training, five reported a decrease in insulin requirement. [11], [13], [15], [22], [23] Those studies with two intervention arms [15], [22] both reported greater insulin dose reductions in the higher intensity arms. However, six studies reported no significant change in insulin dose with their training intervention and only two of these reported a reduction in HbA1c. [16], [24] These studies therefore fail to clarify whether the lack of glycaemic benefit of exercise can be attributed to changes in diet or insulin dose.

HbA1c has been used as a measure of glycaemic control in our analysis but this may not be the most appropriate measure of glycaemic control. To illustrate, glycaemic variability has been suggested to contribute to the development of microvascular complications in type 2 diabetes. [25] Unfortunately, none of the exercise studies in our analysis have examined glycaemic variability. There is recent evidence from patients with insulin treated type 2 diabetes that the time spent in hyperglycaemia is reduced in the 24 hrs following exercise (without an increase in hypoglycaemia), [26]. Conversely in type 1 diabetes, wide blood glucose variability has been reported around exercise in the few studies that have been conducted [27].

In contrast to our results, a recent meta-analysis by Tonoli et al [28] reported a significant but small HbA1c lowering effect of exercise in type 1 diabetes (Cohen’s d −0.27;95% CI −0.06 to −0.47). This paper however used different criteria for study selection. It included studies with no control group, or control subjects without diabetes. We purposefully excluded these trials to control for the effect of participation in a clinical trial. Our meta-analysis also included studies that were excluded in the Tonoli analysis [11], [14], [15], [16], [22], [23], [24]. We believe these differences accounts for the differing conclusions of the two meta-analyses. Tonoli et al [28] did however agree that studies of strength training exercises showed no overall improvement in glycaemic control.

Overall there is a lack of large well-conducted studies on glycaemic benefits of exercise in type 1 diabetes. Our systematic review identified thirteen studies, from which data on 452 patients has been used for analysis. In contrast, a recent meta-analysis of exercise intervention in type 2 diabetes (which did detect an HbA1c lowering effect) analysed 47 RCTs including more than 8500 patients. [4] Further research is therefore required to demonstrate a glycaemic benefit of exercise in type 1 diabetes. We would suggest the following areas are worth considering when designing this research.

1. designing larger trials lasting at least six months
2. trial design that is randomised and well controlled (with matched type 1 diabetic subjects and recording of dietary intake)
3. examining the effect of exercise on glycaemic variability
4. examining the effect of exercise intensity, and the incorporation of a dietary programme on glycaemic benefit
5. examining the effect of age and duration of diabetes on glycaemic benefit

Whilst this meta-analysis did not detect a glycaemic benefit to exercise, there are other well defined benefits in type 1 diabetes. These include reduction in macrovascular risk, mortality, and improvement in wellbeing. [3] Therefore, we suggest exercise should continue to play an important role in the management of type 1 diabetes, whilst its glycaemic benefits are more thoroughly investigated.

## Supporting Information

Figure S1 **Funnel plot testing for publication bias.**
(TIF)

Appendix S1 **Search Strategy.**
(DOCX)
