# Supplementary figures and images for: Does Exercise Improve Glycaemic Control in Type 1 Diabetes? A Systematic Review and Meta-Analysis
Source: PLoS One. 2013 Mar 15;8(3):e58861. doi: 10.1371/journal.pone.0058861 (PMC3598953; doi:10.1371/journal.pone.0058861)

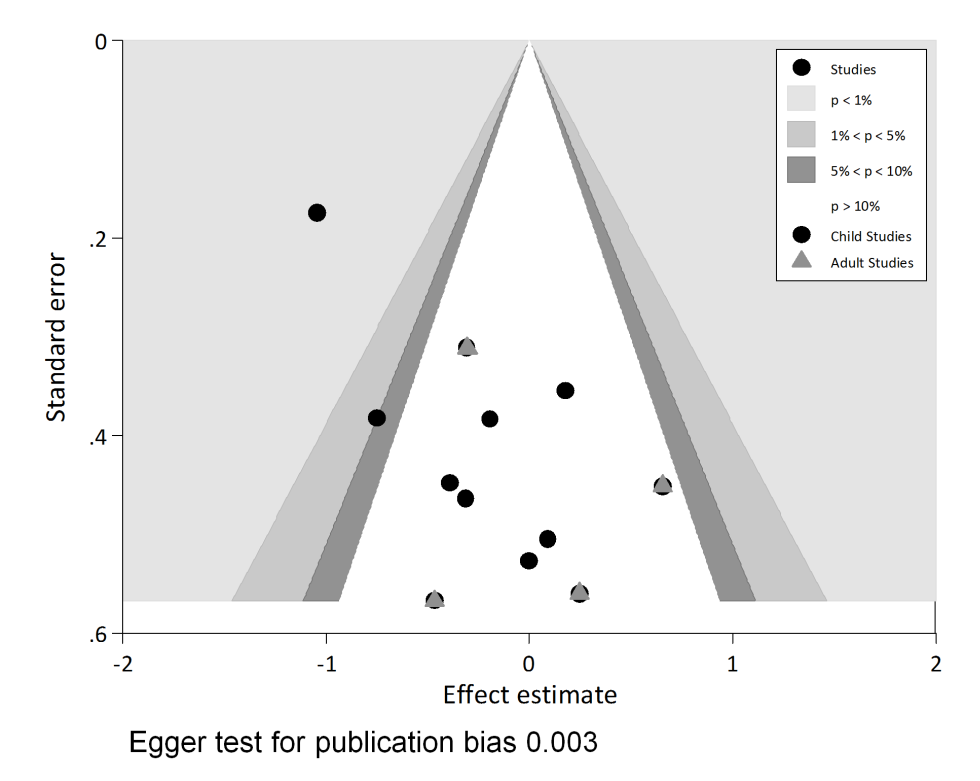

Supplement: Figure S1 — Funnel plot testing for publication bias. (TIF) [file pone.0058861.s001.tif]
