# Supplementary material for: Does Exercise Improve Glycaemic Control in Type 1 Diabetes? A Systematic Review and Meta-Analysis
Source: PLoS One. 2013 Mar 15;8(3):e58861. doi: 10.1371/journal.pone.0058861 (PMC3598953; doi:10.1371/journal.pone.0058861)
Supplement: Appendix S1 — Search Strategy. (DOCX) [file pone.0058861.s002.docx]

**MEDLINE Search**

|  | Searches | Results |
| --- | --- | --- |
| 1 | exp Diabetes Mellitus, Type 1/ or type 1 diabetes.mp. | 58243 |
| 2 | (insulin dependant diabetes mellitus or IDDM).mp. [mp=protocol supplementary concept, rare disease supplementary concept, title, original title, abstract, name of substance word, subject heading word, unique identifier] | 6645 |
| 3 | juvenile diabet$.mp. | 1796 |
| 4 | exp Exercise/ or exercise.mp. | 192002 |
| 5 | physical activity.mp. or exp Motor Activity/ | 127773 |
| 6 | physical activit$.mp. [mp=protocol supplementary concept, rare disease supplementary concept, title, original title, abstract, name of substance word, subject heading word, unique identifier] | 39476 |
| 7 | sports.mp. or exp Sports/ | 110104 |
| 8 | exp Physical Exertion/ or exertion.mp. | 56426 |
| 9 | physical education.mp. or exp "Physical Education and Training"/ | 13276 |
| 10 | exp Recreation/ or recreation.mp. | 108984 |
| 11 | exp Leisure Activities/ or leisure time activity.mp. | 124999 |
| 12 | exp exercise movement techniques/ or exp exercise therapy/ or exp motion therapy, continuous passive/ or exp muscle stretching exercises/ or exp resistance training/ | 27692 |
| 13 | (exercise isometric or aerobic exercise$).mp. [mp=protocol supplementary concept, rare disease supplementary concept, title, original title, abstract, name of substance word, subject heading word, unique identifier] | 3660 |
| 14 | 4 or 5 or 6 or 7 or 8 or 9 or 10 or 11 or 12 or 13 | 397375 |
| 15 | 1 or 2 or 3 | 59421 |
| 16 | 14 and 15 | 2105 |

**EMBASE Search**

| **Searches** | **Results** |
| --- | --- |
| exp insulin dependent diabetes mellitus/ | 62298 |
| type 1 diabetes.mp. | 22193 |
| juvenile diabet$.mp. | 3919 |
| exercise.mp. or exp stretching exercise/ or exp exercise intensity/ or exp muscle exercise/ or exp dynamic exercise/ or exp aquatic exercise/ or exp isokinetic exercise/ or exp leg exercise/ or exp exercise/ or exp anaerobic exercise/ or exp isometric exercise/ or exp aerobic exercise/ or exp static exercise/ or exp isotonic exercise/ | 264437 |
| physical activity.mp. or exp physical activity/ | 188035 |
| sports.mp. or exp sport/ | 102891 |
| physical exertion.mp. | 1800 |
| physical eduction.mp. | 0 |
| recreation.mp. or exp recreation/ | 33983 |
| exp leisure/ or leisure time activity.mp. | 15852 |
| 1 or 2 or 3 | 69256 |
| 4 or 5 or 6 or 7 or 8 or 9 or 10 | 486452 |
| 11 and 12 | 2979 |

**SPORTDiscus**

|  | Query | Results |
| --- | --- | --- |
| S16 | S14 AND S15 | 343 |
| S15 | S3 or S4 or S5 or S6 or S7 or S8 or S9 or S10 or S11 or S12 or s13 | 976796 |
| S14 | S1 or s2 | 799 |
| S13 | DE "SPORTS" OR DE "AERODYNAMICS in sports" OR DE "AERONAUTICAL sports" OR DE "AGE & sports" OR DE "AMATEUR sports" OR DE "ANIMAL sports" OR DE "ANTISEMITISM in sports" OR DE "AQUATIC sports" OR DE "BALL games" OR DE "BALLISTICS in sports" OR DE "BASEBALL" OR DE "BIOMECHANICS in sports" OR DE "CHARITY sports events" OR DE "COLLEGE sports" OR DE "COMMUNICATION in sports" OR DE "CONTACT sports" OR DE "CROSS-training (Sports)" OR DE "DISC golf" OR DE "DISCRIMINATION in sports" OR DE "DOPING in sports" OR DE "ENDURANCE sports" OR DE "EXTREME sports" OR DE "FASCISM & sports" OR DE "FEMINISM & sports" OR DE "GAELIC games" OR DE "GAY Games" OR DE "GAYS & sports" OR DE "GOODWILL Games" OR DE "GROUP games" OR DE "GYMNASTICS" OR DE "HOCKEY" OR DE "HOMOPHOBIA in sports" OR DE "HYDRODYNAMICS in sports" OR DE "INDIVIDUAL sports" OR DE "KINEMATICS in sports" OR DE "KNIFE throwing" OR DE "LESBIANS & sports" OR DE "LGBT people & sports" OR DE "LOG-chopping (Sports)" OR DE "MASCULINITY in sports" OR DE "MASS media & sports" OR DE "MILITARY sports" OR DE "MINORITIES in sports" OR DE "MOTION pictures in sports" OR DE "MOTORSPORTS" OR DE "NATIONAL socialism & sports" OR DE "NATIONALISM & sports" OR DE "OLYMPICS" OR DE "PARKOUR" OR DE "PHOTOGRAPHY of sports" OR DE "PHYSICS in sports" OR DE "PRESIDENTS -- Sports" OR DE "PRESIDENTS -- Sports -- United States" OR DE "PROFESSIONAL sports" OR DE "PROFESSIONALISM in sports" OR DE "RACING" OR DE "RACISM in sports" OR DE "RACKET games" OR DE "RECREATIONAL sports" OR DE "REGIONALISM & sports" OR DE "ROBOTICS in sports" OR DE "RODEOS" OR DE "ROLLER skating" OR DE "SCHOOL sports" OR DE "SENIOR Olympics" OR DE "SEX discrimination in sports" OR DE "SEXUAL harassment in sports" OR DE "SHOOTING" OR DE "SHUTOUTS (Sports)" OR DE "SOCIALISM & sports" OR DE "SPORT for All" OR DE "SPORTS & tourism" OR DE "SPORTS -- Collectibles" OR DE "SPORTS -- Corrupt practices" OR DE "SPORTS -- Economic aspects" OR DE "SPORTS -- Finance" OR DE "SPORTS -- Folklore" OR DE "SPORTS -- Songs & music" OR DE "SPORTS for children" OR DE "SPORTS for people with disabilities" OR DE "SPORTS for women" OR DE "SPORTS forecasting" OR DE "SPORTS in antiquity" OR DE "SPORTS in video games" OR DE "SPORTS penalties" OR DE "SPORTS rivalries" OR DE "SPORTS teams" OR DE "STEREOTYPES (Social psychology) in sports" OR DE "TARGETS (Sports)" OR DE "TEAMWORK (Sports)" OR DE "TELEVISION & sports" OR DE "TOMAHAWK throwing" OR DE "TRACEURS" OR DE "VIDEO tapes in sports" OR DE "VIOLENCE in sports" OR DE "WINTER sports" | 249076 |
| S12 | (DE "PHYSICAL activity" OR DE "PHYSICAL education -- Moral & ethical aspects") | 2147 |
| S11 | DE "EXERCISE" OR DE "ABDOMINAL exercises" OR DE "AEROBIC exercises" OR DE "ANAEROBIC exercises" OR DE "AQUATIC exercises" OR DE "ARM exercises" OR DE "BACK exercises" OR DE "BREATHING exercises" OR DE "BREEMA" OR DE "BUTTOCKS exercises" OR DE "CALISTHENICS" OR DE "CHAIR exercises" OR DE "CIRCUIT training" OR DE "COMPOUND exercises" OR DE "DO-in" OR DE "EXERCISE -- Immunological aspects" OR DE "EXERCISE adherence" OR DE "EXERCISE therapy" OR DE "FACIAL exercises" OR DE "FALUN gong exercises" OR DE "GYMNASTICS" OR DE "HAND exercises" OR DE "HATHA yoga" OR DE "ISOKINETIC exercise" OR DE "ISOLATION exercises" OR DE "ISOMETRIC exercise" OR DE "ISOTONIC exercise" OR DE "LEG exercises" OR DE "LIANGONG" OR DE "METABOLIC equivalent" OR DE "MULAN quan" OR DE "MUSCLE strength" OR DE "PHYSICAL fitness for men" OR DE "PILATES method" OR DE "PLYOMETRICS" OR DE "QI gong" OR DE "REDUCING exercises" OR DE "RUNNING" OR DE "RUNNING -- Social aspects" OR DE "SCHOOLS -- Exercises & recreations" OR DE "SEXUAL exercises" OR DE "SHOULDER exercises" OR DE "STRENGTH training" OR DE "STRETCHING exercises" OR DE "TAI chi" OR DE "TREADMILL exercise" OR DE "WHEELCHAIR workouts" OR DE "YANTRA yoga" OR DE "EXERCISE for children" OR DE "EXERCISE for men" OR DE "PHYSICAL fitness for men" | 129662 |
| S10 | TX physical exertion or physical education or recreation or leisure time activit* | 170197 |
| S9 | SU physical exertion or physical education or recreation or leisure time activit* | 85745 |
| S8 | SU sports | 338587 |
| S7 | TX sport* | 771926 |
| S6 | SU physical activity | 2188 |
| S5 | TX physical activity | 34529 |
| S4 | SU exercise | 107979 |
| S3 | TX exercise | 166586 |
| S2 | DE "ACETONEMIA" OR DE "DIABETES in children" OR DE "DIABETES in youth" | 123 |
| S1 | TX type 1 diabet* or T1DM or IDDM or insulin dependent diabetes mellitus or juvenile diabet* or insulin dependant diabetes mellitus | 724 |

**COCHRANE LIBRARY**

| **ID** | **Search** | **Hits** | **Edit** | **Delete** |
| --- | --- | --- | --- | --- |
| #1 | [type 1 diabetes](http://onlinelibrary.wiley.com/o/cochrane/searchHistory?mode=runquery&qnum=1) | 18775 | [edit](JavaScript:doPopup('/search-web/cochrane/searchHistory?mode=editquery&qnum=1&searchKey=d7dd9f7f-f052-4f0f-ad37-1cad1551f046',%20400)) | [delete](http://onlinelibrary.wiley.com/search-web/cochrane/searchHistory?mode=deletequery&qnum=1&uuid=d7dd9f7f-f052-4f0f-ad37-1cad1551f046&searchKey=d7dd9f7f-f052-4f0f-ad37-1cad1551f046) |
| #2 | [MeSH descriptor **Diabetes Mellitus, Type 1** explode all trees](http://onlinelibrary.wiley.com/o/cochrane/searchHistory?mode=runquery&qnum=2) | 2763 | [edit](http://onlinelibrary.wiley.com/search-web/cochrane/searchHistory?mode=editquery&qnum=2&searchKey=d7dd9f7f-f052-4f0f-ad37-1cad1551f046) | [delete](http://onlinelibrary.wiley.com/search-web/cochrane/searchHistory?mode=deletequery&qnum=2&uuid=d7dd9f7f-f052-4f0f-ad37-1cad1551f046&searchKey=d7dd9f7f-f052-4f0f-ad37-1cad1551f046) |
| #3 | [NIDDM OR non insulin dependant diabetes mellitus](http://onlinelibrary.wiley.com/o/cochrane/searchHistory?mode=runquery&qnum=3) | 1045 | [edit](JavaScript:doPopup('/search-web/cochrane/searchHistory?mode=editquery&qnum=3&searchKey=d7dd9f7f-f052-4f0f-ad37-1cad1551f046',%20400)) | [delete](http://onlinelibrary.wiley.com/search-web/cochrane/searchHistory?mode=deletequery&qnum=3&uuid=d7dd9f7f-f052-4f0f-ad37-1cad1551f046&searchKey=d7dd9f7f-f052-4f0f-ad37-1cad1551f046) |
| #4 | [juvenile diabetes](http://onlinelibrary.wiley.com/o/cochrane/searchHistory?mode=runquery&qnum=4) | 84 | [edit](JavaScript:doPopup('/search-web/cochrane/searchHistory?mode=editquery&qnum=4&searchKey=d7dd9f7f-f052-4f0f-ad37-1cad1551f046',%20400)) | [delete](http://onlinelibrary.wiley.com/search-web/cochrane/searchHistory?mode=deletequery&qnum=4&uuid=d7dd9f7f-f052-4f0f-ad37-1cad1551f046&searchKey=d7dd9f7f-f052-4f0f-ad37-1cad1551f046) |
| #5 | [(#1 OR #2 OR #3 OR #4)](http://onlinelibrary.wiley.com/o/cochrane/searchHistory?mode=runquery&qnum=5) | 18935 | [edit](JavaScript:doPopup('/search-web/cochrane/searchHistory?mode=editquery&qnum=5&searchKey=d7dd9f7f-f052-4f0f-ad37-1cad1551f046',%20400)) | [delete](http://onlinelibrary.wiley.com/search-web/cochrane/searchHistory?mode=deletequery&qnum=5&uuid=d7dd9f7f-f052-4f0f-ad37-1cad1551f046&searchKey=d7dd9f7f-f052-4f0f-ad37-1cad1551f046) |
| #6 | [exercise](http://onlinelibrary.wiley.com/o/cochrane/searchHistory?mode=runquery&qnum=6) | 35329 | [edit](JavaScript:doPopup('/search-web/cochrane/searchHistory?mode=editquery&qnum=6&searchKey=d7dd9f7f-f052-4f0f-ad37-1cad1551f046',%20400)) | [delete](http://onlinelibrary.wiley.com/search-web/cochrane/searchHistory?mode=deletequery&qnum=6&uuid=d7dd9f7f-f052-4f0f-ad37-1cad1551f046&searchKey=d7dd9f7f-f052-4f0f-ad37-1cad1551f046) |
| #7 | [MeSH descriptor **Exercise** explode all trees](http://onlinelibrary.wiley.com/o/cochrane/searchHistory?mode=runquery&qnum=7) | 8169 | [edit](http://onlinelibrary.wiley.com/search-web/cochrane/searchHistory?mode=editquery&qnum=7&searchKey=d7dd9f7f-f052-4f0f-ad37-1cad1551f046) | [delete](http://onlinelibrary.wiley.com/search-web/cochrane/searchHistory?mode=deletequery&qnum=7&uuid=d7dd9f7f-f052-4f0f-ad37-1cad1551f046&searchKey=d7dd9f7f-f052-4f0f-ad37-1cad1551f046) |
| #8 | [physical activity](http://onlinelibrary.wiley.com/o/cochrane/searchHistory?mode=runquery&qnum=8) | 10366 | [edit](JavaScript:doPopup('/search-web/cochrane/searchHistory?mode=editquery&qnum=8&searchKey=d7dd9f7f-f052-4f0f-ad37-1cad1551f046',%20400)) | [delete](http://onlinelibrary.wiley.com/search-web/cochrane/searchHistory?mode=deletequery&qnum=8&uuid=d7dd9f7f-f052-4f0f-ad37-1cad1551f046&searchKey=d7dd9f7f-f052-4f0f-ad37-1cad1551f046) |
| #9 | [MeSH descriptor **Physical Education and Training** explode all trees](http://onlinelibrary.wiley.com/o/cochrane/searchHistory?mode=runquery&qnum=9) | 1218 | [edit](http://onlinelibrary.wiley.com/search-web/cochrane/searchHistory?mode=editquery&qnum=9&searchKey=d7dd9f7f-f052-4f0f-ad37-1cad1551f046) | [delete](http://onlinelibrary.wiley.com/search-web/cochrane/searchHistory?mode=deletequery&qnum=9&uuid=d7dd9f7f-f052-4f0f-ad37-1cad1551f046&searchKey=d7dd9f7f-f052-4f0f-ad37-1cad1551f046) |
| #10 | [MeSH descriptor **Leisure Activities** explode all trees](http://onlinelibrary.wiley.com/o/cochrane/searchHistory?mode=runquery&qnum=10) | 8102 | [edit](http://onlinelibrary.wiley.com/search-web/cochrane/searchHistory?mode=editquery&qnum=10&searchKey=d7dd9f7f-f052-4f0f-ad37-1cad1551f046) | [delete](http://onlinelibrary.wiley.com/search-web/cochrane/searchHistory?mode=deletequery&qnum=10&uuid=d7dd9f7f-f052-4f0f-ad37-1cad1551f046&searchKey=d7dd9f7f-f052-4f0f-ad37-1cad1551f046) |
| #11 | [leisure activit*](http://onlinelibrary.wiley.com/o/cochrane/searchHistory?mode=runquery&qnum=11) | 572 | [edit](JavaScript:doPopup('/search-web/cochrane/searchHistory?mode=editquery&qnum=11&searchKey=d7dd9f7f-f052-4f0f-ad37-1cad1551f046',%20400)) | [delete](http://onlinelibrary.wiley.com/search-web/cochrane/searchHistory?mode=deletequery&qnum=11&uuid=d7dd9f7f-f052-4f0f-ad37-1cad1551f046&searchKey=d7dd9f7f-f052-4f0f-ad37-1cad1551f046) |
| #12 | [MeSH descriptor **Sports** explode all trees](http://onlinelibrary.wiley.com/o/cochrane/searchHistory?mode=runquery&qnum=12) | 6748 | [edit](http://onlinelibrary.wiley.com/search-web/cochrane/searchHistory?mode=editquery&qnum=12&searchKey=d7dd9f7f-f052-4f0f-ad37-1cad1551f046) | [delete](http://onlinelibrary.wiley.com/search-web/cochrane/searchHistory?mode=deletequery&qnum=12&uuid=d7dd9f7f-f052-4f0f-ad37-1cad1551f046&searchKey=d7dd9f7f-f052-4f0f-ad37-1cad1551f046) |
| #13 | [sports](http://onlinelibrary.wiley.com/o/cochrane/searchHistory?mode=runquery&qnum=13) | 8709 | [edit](JavaScript:doPopup('/search-web/cochrane/searchHistory?mode=editquery&qnum=13&searchKey=d7dd9f7f-f052-4f0f-ad37-1cad1551f046',%20400)) | [delete](http://onlinelibrary.wiley.com/search-web/cochrane/searchHistory?mode=deletequery&qnum=13&uuid=d7dd9f7f-f052-4f0f-ad37-1cad1551f046&searchKey=d7dd9f7f-f052-4f0f-ad37-1cad1551f046) |
| #14 | [sport*](http://onlinelibrary.wiley.com/o/cochrane/searchHistory?mode=runquery&qnum=14) | 9655 | [edit](JavaScript:doPopup('/search-web/cochrane/searchHistory?mode=editquery&qnum=14&searchKey=d7dd9f7f-f052-4f0f-ad37-1cad1551f046',%20400)) | [delete](http://onlinelibrary.wiley.com/search-web/cochrane/searchHistory?mode=deletequery&qnum=14&uuid=d7dd9f7f-f052-4f0f-ad37-1cad1551f046&searchKey=d7dd9f7f-f052-4f0f-ad37-1cad1551f046) |
| #15 | [recreation](http://onlinelibrary.wiley.com/o/cochrane/searchHistory?mode=runquery&qnum=15) | 501 | [edit](JavaScript:doPopup('/search-web/cochrane/searchHistory?mode=editquery&qnum=15&searchKey=d7dd9f7f-f052-4f0f-ad37-1cad1551f046',%20400)) | [delete](http://onlinelibrary.wiley.com/search-web/cochrane/searchHistory?mode=deletequery&qnum=15&uuid=d7dd9f7f-f052-4f0f-ad37-1cad1551f046&searchKey=d7dd9f7f-f052-4f0f-ad37-1cad1551f046) |
| #16 | [MeSH descriptor **Recreation** explode all trees](http://onlinelibrary.wiley.com/o/cochrane/searchHistory?mode=runquery&qnum=16) | 7143 | [edit](http://onlinelibrary.wiley.com/search-web/cochrane/searchHistory?mode=editquery&qnum=16&searchKey=d7dd9f7f-f052-4f0f-ad37-1cad1551f046) | [delete](http://onlinelibrary.wiley.com/search-web/cochrane/searchHistory?mode=deletequery&qnum=16&uuid=d7dd9f7f-f052-4f0f-ad37-1cad1551f046&searchKey=d7dd9f7f-f052-4f0f-ad37-1cad1551f046) |
| #17 | [(**#6** OR **#7** OR **#8** OR **#9** OR **#10** OR **#11** OR **#12** OR **#13** OR **#14** OR **#15#** OR **#16**)](http://onlinelibrary.wiley.com/o/cochrane/searchHistory?mode=runquery&qnum=17) | 46313 | [edit](JavaScript:doPopup('/search-web/cochrane/searchHistory?mode=editquery&qnum=17&searchKey=d7dd9f7f-f052-4f0f-ad37-1cad1551f046',%20400)) | [delete](http://onlinelibrary.wiley.com/search-web/cochrane/searchHistory?mode=deletequery&qnum=17&uuid=d7dd9f7f-f052-4f0f-ad37-1cad1551f046&searchKey=d7dd9f7f-f052-4f0f-ad37-1cad1551f046) |
| #18 | [(#5 AND #17)](http://onlinelibrary.wiley.com/o/cochrane/searchHistory?mode=runquery&qnum=18) | 2312 | [edit](JavaScript:doPopup('/search-web/cochrane/searchHistory?mode=editquery&qnum=18&searchKey=d7dd9f7f-f052-4f0f-ad37-1cad1551f046',%20400)) | [delete](http://onlinelibrary.wiley.com/search-web/cochrane/searchHistory?mode=deletequery&qnum=18&uuid=d7dd9f7f-f052-4f0f-ad37-1cad1551f046&searchKey=d7dd9f7f-f052-4f0f-ad37-1cad1551f046) |
